# Sociocultural predictors of immigrant adjustment and well-being

**DOI:** 10.3389/fsoc.2024.1251871

**Published:** 2024-02-29

**Authors:** Ia Shekriladze, Nino Javakhishvili

**Affiliations:** D. Uznadze Institute of Psychology, Ilia State University, Tbilisi, Georgia

**Keywords:** immigration, sociocultural adjustment, psychological adjustment, depression, perceived discrimination, language fluency, intercultural distance

## Abstract

**Introduction:**

Research shows that culture change may pose risks to immigrant wellbeing. Our study examined adult Georgians (*N* = 431) residing in Greece, Italy, and Germany, and explored associations between their demographic characteristics, sociocultural adjustment, and psychological well-being outcomes.

**Methods:**

Conducted via electronic self-report survey, the cross-sectional study measured participants’ levels of sociocultural adjustment, psychological adjustment, and depression along with the willingness to interact with host nationals, perceived sense of discrimination, history of being undocumented, age and length of relocation, and fluency in host language. The study also examined differences in three subsamples from the standpoint of intercultural distance. Sociocultural Adjustment Scale, Brief Psychological Adaptation Scale, Center for Epidemiologic Studies Depression Scale and Host Interaction Scale were used to measure the corresponding variables. Perceived history of discrimination was measured by a Likert-scale question about discrimination in a host country. Intercultural distance was established by Hofstede cultural compass and was estimated to be the smallest with Greece and the largest with Germany.

**Results:**

Depression was positively predicted by histories of discrimination and illegal immigration, host language fluency upon relocation, and was negatively predicted by sociocultural adjustment. Psychological adjustment was positively predicted by sociocultural adjustment, willingness to interact with host nationals, and ongoing language fluency, while perceived sense of discrimination, age, and poor financial state acted as negative predictors. Finally, sociocultural adjustment acted as the strongest determinant of wellbeing predicting both lower depression and higher psychological adjustment.

**Discussion:**

Our findings suggested that adjustment in diverse sociocultural domains was the most critical for the immigrants’ psychological well-being along with the lack of perceived discrimination. Additional factors associated with the better adaptation outcomes included younger age, willingness to interact with host nationals, language fluency, better financial standing and no history of being undocumented. The results also indicated that host language proficiency upon relocation may contribute to migrant susceptibility, whereas intercultural distance may be overshadowed in importance by acculturation conditions. The findings illustrate the complexity of migration and culture change and point to the superiority of wholistic policies and practices in promoting smooth transition of immigrant populations.

## 1 Introduction

Research on immigrant well-being indicates that cross-cultural transition may pose significant risks to individual psychological functioning. A long-term relocation outside of one’s country of origin entails exposure to multiple drastic changes along with the lack of certainty about the new life in a new culture. Thus, culture change is accompanied with acculturative stress related to the migratory experience and adjustment to the host country, which is regarded to play a crucial role in immigrants’ mental health (Ward and Geeraert, 2016).

Multiple studies conducted on psychological well-being of immigrant populations have confirmed the vulnerability of migrants (especially refugees and asylum seekers) to major mental health issues such as depression, anxiety and posttraumatic stress disorder (Toselli et al., 2014; George et al., 2015; Bustamante et al., 2017; Martin and Sashidharan, 2023). Access to mental healthcare, on the other hand, has been reported to be an issue for migrants due to variety of reasons including language barrier and negative attitudes towards them (Satinsky et al., 2019). Undocumented migrants particularly tend to fall outside of the existing healthcare and social services in many European countries (Lindert et al., 2008; Woodward et al., 2014; Winters et al., 2018).

Acculturation has been defined as a process of adopting values, beliefs, behaviors, language and identities of another culture as a result of being exposed to it (Berry, 2006; Ward and Geeraert, 2016). Prominent bidimensional model of acculturation conceptualized individual’s home culture orientation and host culture orientation as independent dimensions (Berry, 2006). Subsequent evidence from immigrant studies suggested that acculturation strategy of integration/biculturalism—higher levels of both home culture and host culture orientations—was linked to better adaptation and psychological functioning compared to the engagement in one culture only, while lack of interest in either culture was associated with the poorest outcomes (Berry, 2006; Sam and Berry, 2010; Celenk and Van de Vijver, 2011; Ward and Geeraert, 2016).

Some studies, however, produced evidence supporting higher importance of host culture orientation in immigrant adjustment compared to home culture orientation. Results of these studies indicated that individuals with assimilation strategy of acculturation (high host culture orientation and low home culture orientation) produced as good well-being outcomes as integrated individuals (Birman et al., 2002; Berry and Hou, 2014; Ward et al., 2017). In some studies, higher mainstream culture orientation was linked with better well-being outcomes irrespective of home culture orientation (Schachner et al., 2017). These findings pinpointed the superior role of engagement in the society of residence and building social connections with host culture in immigrant adaptation.

Ward and colleagues emphasized the roles of psychological and sociocultural adaptation in the process of acculturation (Searle and Ward, 1990; Ward, 2001). A 2013 meta-analysis of 83 studies reported integration strategy of acculturation to be strongly and positively associated with both psychological and sociocultural adjustment (Nguyen and Benet-Martínez, 2013). These findings suggested crucial role of psychological and sociocultural adjustment in immigrant integration.

Depression has also been researched extensively and linked with migrant psychological and sociocultural adjustment. A study on Chinese migrant youth showed psychological adjustment to be directly affected by symptoms of depression as well as social interaction and fluency in language (Shi et al., 2019). Social support and sociocultural adjustment were identified as predictors of the level of depressiveness by another study with social support being singled out as an independent predictor of well-being (Razgulin et al., 2023).

Apart from specific individual contexts that prompt people to embark on the journey of migration, variety of external factors shape their experiences of cross-cultural transition. These characteristics are called acculturation conditions and entail antecedent and concurrent individual, situational and social factors, home-society, and host-society characteristics (Arends-Tóth and Van de Vijver, 2006; Celenk and Van de Vijver, 2011). Individual-level acculturation conditions include such characteristics as personality features, expectations to a new country, language fluency, and goal of relocation. Group-level acculturation conditions involve socioeconomic and political contexts of the home country (e.g., forced migration due to war or extreme poverty) and the host country (e.g., perceived discrimination, immigration policies).

Abundance of evidence supports an important role of acculturation conditions in migrants’ adaptation. Legal immigration status, social contacts, employability, fluency in a host language, among others, have been linked with better adjustment and behavioral health outcomes (Kosic, 2004; Arends-Tóth and Van de Vijver, 2006; George et al., 2015). Social support and fluency in English were identified as important predictors of better adaptation by studies from variety of countries (Ozer, 2015; Darmanaki Farahani and Bradley, 2018; Pinamang et al., 2021; Mohanan, 2022). Connections with host nationals was singled out as an important factor for psychological adaptation of foreign students mediating the effect of language proficiency on adaptive outcomes (Bethel et al., 2020).

Evidence suggests that the subjective sense of being discriminated against is linked to poorer adaptation and mental well-being outcomes among immigrants. Perceived discrimination was reported an important barrier to adjustment for international students in various countries (Ozer, 2015; Adger, 2016). Studies of Venezuelan migrants in Peru (Mougenot et al., 2021), as well as in Colombia and the US (Schwartz et al., 2018), suggested that perceived discrimination considerably increased the risk of mental health problems. Another study from Spain confirmed the link between the perceived discrimination and psychological distress among immigrants pinpointing the moderating role of social network in reducing the negative effect of perceived discrimination on mental health (García-Cid et al., 2020). Christ and colleagues examined prejudice and acculturation preferences of native Germans and discovered that a negative climate of reception predicted a weaker desire for embracing new culture among immigrants (Christ et al., 2015; Sam and Ward, 2021).

In addition, intercultural distance between the original culture and host culture is regarded as an important factor in cross-cultural transition (Searle and Ward, 1990; Berry, 1992). Hofstede culture compass offers country profiles on the basis of the following dimensions: power distance (strength of social hierarchy), individualism, masculinity (task-orientation versus person-orientation), uncertainty avoidance, long term orientation, and indulgence (Hofstede Insights, n.d.). Multiple studies have suggested that proximity between home and host cultures eases immigrant psychological and sociocultural adaptation (Nesdale and Mak, 2003; Galchenko and Van de Vijver, 2007), unless they are received negatively or discriminated against (Schwartz et al., 2018).

Thus, psychological, and sociocultural adaptation and mental well-being along with the key acculturation conditions and intercultural distance represent important variables to examine in research of immigrant populations. Over the last decades, acculturation psychology has grown tremendously producing abundance of new evidence (Sam and Ward, 2021), which speaks volumes about the ever-growing need for understanding the complexity of cross-cultural transition.

### 1.1 The present study

Beginning from 1990s after country’s regaining independence, the number of individuals emigrating from Georgia in search of better prospects rose consistently. According to the State Commission on Migration Issues (2021), over the last decades, the number of Georgian citizens holding valid residence permits in EU/Schengen States kept increasing and rose by 25% in 2019. Over 50% of Georgians holding valid residence permits in EU/Schengen States reside in Greece, Italy and Germany. Among immigrant populations of Georgia, women considerably outnumber men and tend to be older; female labor migration is especially common in Italy and Greece with growing market of domestic work and eldercare (Hofmann and Buckley, 2013; Vanore and Siegel, 2015; Adger, 2016; Huss and Frelak, 2019; Zurabishvili, 2022). According to the National Statistics Office of Georgia (2014), female to male migration ratio in Italy is six to one, in Greece – almost five to one, as caretaking jobs are offered primarily to women resulting in feminization of labor migration, whereas per Italy’s National Agency for Active Labour Policies (ANPAL), women comprised 82% of all Georgians residing in Italy (Ministero del lavoro e delle politiche sociali, 2021, p. 14, as cited in Zurabishvili, 2022) having the highest female share among all ethnic groups.

Despite high numbers of Georgians leaving the country, studies on their well-being outcomes are very limited. A study on Georgian women residing in the US and UK showed that high host culture orientation and history of marriage with host national predicted healthier eating patterns (Shekriladze et al., 2019). Delayed integration in mainstream culture was identified among Georgians residing in Portugal (Pirtskhalava, 2017), while a recent qualitative study on Georgian migrants in EU and USA pinpointed low levels of their integration and sociocultural adaptation (Pirtskhalava and Badashvili, 2022).

The present study examined adult Georgians residing in three EU countries: Greece, Italy, and Germany. The aim of the study was to explore the associations among the immigrant demographic characteristics, acculturation conditions, sociocultural and psychological adjustment and depression. Variables such as age at relocation, lengths of residence, history of being undocumented, history of marriage with host national, willingness to interact with host nationals, perceived sense of discrimination, frequency of visiting homeland, fluency of host language upon relocation and at the time of the study, were examined.

Since the measures of psychological adjustment and depression entailed assessing one’s state during the last two weeks, while assessment of sociocultural adjustment required from the participant to recall how his or her adjustment evolved throughout the process of relocation, we conceptualized depression and psychological adjustment as outcome variables and sociocultural adjustment and other antecedent and concurrent factors (e.g., age, length of residence, age at relocation, fluency of language, perceived sense of discrimination, etc.) as predictor variables.

We hypothesized the length of residence, fluency in host language, history of marriage with host national, sociocultural adjustment, and willingness to interact with host culture to positively predict psychological adjustment and negatively predict depression. We also presumed age at relocation, history of being undocumented, and perceived sense of discrimination to negatively predict psychological adjustment and positively predict depression.

**Figure 1:**
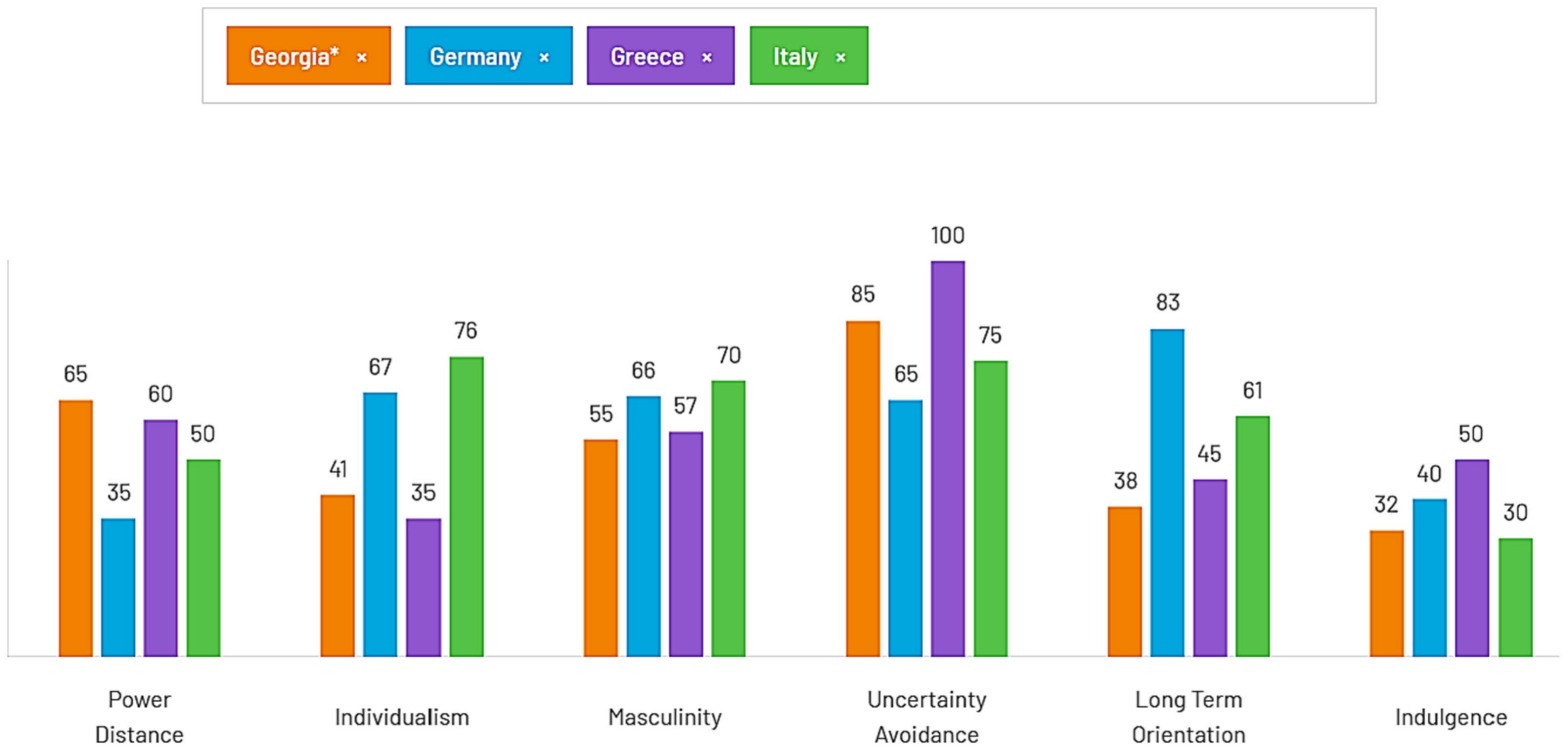
Hofstede insight country comparison (Georgia, Germany, Greece, Italy).

Furthermore, since the study examined immigrant populations in three different cultures, we also aimed to explore whether the values of the main study variables varied depending on the country of residence and in any way associated with the proximity/distance between the host and home culture characteristics. According to the Hofstede country comparison tool (Hofstede Insights, n.d.), the profile of Georgia is closest to the profile of Greece: in four out of six dimensions, scores of these two cultures are most approximate (65 and 60 in power distance, 41 and 35 in individualism, 55 and 57 in masculinity, and 38 and 45 in long term orientation) in comparison with the other two countries (see Figure 1). The other two dimensions are indulgence and uncertainty avoidance in which the scores of Georgia and Italy come closest (32 and 30; 85 and 75 respectively). Thus, overall the cultural distance can be regarded largest with Germany and smallest with Greece and, roughly, adjustment and well-being outcomes of Georgian immigrants can be expected to mirror intercultural proximity.

## 2 Materials and methods

### 2.1 Participants and procedure

The study measured participants’ levels of sociocultural adjustment, psychological adjustment, and depression. Among demographic variables, age, employment status, age at relocation, length of residence, marital status, current financial status, perceived sense of fluency of host language at the time of the study and upon relocation, history of being married to a host national, and history of being undocumented were explored. Participants’ willingness to socialize with host nationals was also studied along with the perceived sense of discrimination in a host country.

A cross-sectional study examined a convenience sample of 431 Georgians, aged 18–65, residing in Greece, Italy, and Germany. The sample comprised of ethnically Georgian individuals, born and raised in Georgia with Georgian being their native language, who had been residing in respective countries for at least 6 months. Participants completed an anonymous online survey consisting of self-report questionnaires as well as questions about demographic and other socio-cultural factors. The survey link included the goal of a study, criteria for participation, an approximate time of its completion as well as a disclaimer about the anonymity of the survey. Ethics approval (R/09422) was obtained from the Ethics Committee of Ilia State University.

Social media and other electronic means of communication were extensively used to reach potential participants. Consular sections of corresponding countries were asked to circulate the study link among their groups. The link was extensively posted in immigrant groups on social media and participants were asked to circulate the study link among their contacts.

The majority of the sample comprised women (93%) with mean age of 43. In total, 201 participants resided in Germany, 155—in Italy, and 75—in Greece. A large part of participants was married (47%), employed full-time (61%), and reported satisfactory financial state (46%). The mean length of residence in a new country for the sample equaled to 10 years. One third of the participants reported visiting home country on average once a year. About one third reported a history of being undocumented (see Table 1).

**Table 1:** Sample demographics.

		**%**
Country	Italy	35.96
	Greece	17.40
	Germany	46.64
Gender	Female	93.97
	Male	6.03
Marital status	Single	27.67
	Married	46.98
	Divorced	15.35
	Widow	8.37
	Other	1.63
Employment	Full-time	61.3
	Part-time	11.8
	Student	8.1
	Housewife	10.4
	Retired/other	3
	Unemployed	4.4
Financial state	Insufficient income	7.19
	Income sufficient only for basic needs	25.06
	Satisfactory	46.40
	Good	18.33
	Excellent	3.02
History of illegal status	No	68.91
	Yes	31.09
History of marriage with host nationals	No	84.22
	Yes	15.78
Age	32	25.29
	42	22.97
	52	26.68
	62	21.35
	+62	3.72
Length of residence	5	36.34
	10	25.77
	15	16.24
	20	10.05
	25	7.99
	+25	3.61
Frequency of visiting Georgia	Not at all	29.23
	Once in 4+ years	11.37
	Once in 2–3 years	16.24
	Once in a year	33.18
	Twice or more in a year	9.98

### 2.2 Measures

*Sociocultural adjustment* was measured by the 12-item Sociocultural Adjustment Scale (Demes and Geeraert, 2014) measuring individual’s sense of adaptation to various life domains in a host culture. These domains included climate, natural environment, social environment, daily practicalities, host language, making friends, social attitudes and norms, family life, standard of living, among others. A 5-point Likert scale from “very difficult” to “very easy” was used to measure adaptation. Higher scores indicated higher adaptation. The Georgian version of the scale was validated via confirmatory factor analysis (CFA) in MPlus, version 6.12. We checked the model for one latent factor: The model fit indices became satisfactory after adding some inter-item correlations: *χ*^2^(31) = 101.97, *p* < 0.001, RMSEA = 0.067, CFI = 0.931, TLI = 0.901, SRMR = 0.046, all variable loadings were above 0.41.

*Psychological adjustment* was measured by the 8-item Brief Psychological Adaptation Scale (BPAS; Demes and Geeraert, 2014) measuring individual’s positive and negative emotional responses to the host culture (e.g., “excited about being in a host country,” “sad about being away from home country”). A 5-point Likert scale from “fully disagree” to “fully agree” was used to measure adaptation. Some items required reversed coding. Higher scores indicated higher adaptation. The Georgian version of the scale was validated via confirmatory factor analysis (CFA) in MPlus, version 6.12. We checked the model for one latent factor: The model fit indices were all good: *χ*^2^(17) = 49.77, *p* < 0.001, RMSEA = 0.062, CFI = 0.971, TLI = 0.953, SRMR = 0.038, all variable loadings were above 0.45.

*Level of depression* was measured by the 8-item Center for Epidemiologic Studies Depression Scale (Irwin et al., 1999). The tool measures individual’s state of mood during the last two-weeks (e.g., “I felt everything I did was an effort”; “I felt lonely”). A 4-point Likert scale from “fully disagree” to “fully agree” was used. Higher scores indicated higher levels of depression. The instrument has been previously validated for Georgian population (Javakhishvili et al., 2016).

*Willingness to socialize with host nationals* was measured by the 3-item Host Interaction Scale that was composed of items borrowed from Vancouver Index of Acculturation (Ryder et al., 2000). The instrument has been validated for Georgian population (Javakhishvili et al., 2016). These items included: “I enjoy social activities with host people,” “I am comfortable interacting with people of host culture,” and “I am interested in having friends of the host culture.” A 5-point Likert scale from “fully disagree” to “fully agree” was used. Higher scores indicated higher willingness to interact.

*Perceived history of discrimination* was measured by a single item question about the experience of being discriminated against with 5-point Likert scale ranging from “almost never” to “very often”.

### 2.3 Statistical analysis

We analyzed data using the statistical package IBM SPSS version 21.00. We calculated descriptive statistics and used ANOVA and chi-square (*χ*^2^) to examine statistically significant differences among subsamples per country of residence. We performed correlational analyses using Pearson’s *r* coefficient, and regression analyses to identify predictors. A probability level of 0.05 was applied in all statistical tests of significance. CFA in MPlus was performed with two scales used for the first time with Georgian population.

## 3 Results

### 3.1 Descriptive data

#### 3.1.1 Overall sample

The sample’s mean scores of psychological and sociocultural adjustments as well as willingness to socialize with the host people were above scale’s midpoint, whereas the mean scores of depression and perceived sense of discrimination were below midpoint, the latter being particularly low. Besides, the mean score for language fluency at the time of the study was far higher (and considerably above midpoint) than the mean score of language fluence upon relocation (see Table 2).

**Table 2:** Sample means, SD and correlations.

	M	SD	1	2	3	4.	5	6	7	8	9
1 Length of residence	9.82	7.60									
2 Age	42.76	11.49	0.38**								
3 Fluency of host language upon relocation	1.83	1.14	−0.00	−0.25**							
4 Fluency of host language now	3.94	0.92	0.45**	−0.17**	0.42**						
5 Frequency of visiting Georgia	2.82	1.42	0.44**	−0.09	0.29**	0.47**					
6 Perceived sense of discrimination	1.99	1.02	−0.05	−0.04	−0.02	−0.05	−0.09				
7 Depression	2.10	0.71	−0.17**	0.09	−0.06	−0.24**	−0.24**	0.26**			
8 Willingness to interact with host nationals	3.67	0.78	0.10*	−0.19**	0.11*	0.24**	0.22**	−0.31**	−0.20**		
9 Sociocultural adjustment	3.76	0.65	0.16**	−0.02	0.13**	0.18**	0.19**	−0.37**	−0.34**	0.33**	
10 Psychological adjustment	3.18	0.86	0.14**	−0.22**	0.20**	0.34**	0.27**	−0.41**	−0.57**	0.37**	0.46**

**p ≤ 0.001.

### 3.2 Correlations

Correlational analyses revealed significant links among multiple variables (see Table 2). Psychological and sociocultural adaption positively correlated (significant moderate correlation) with one another, while the strongest yet negative correlations were identified between psychological adjustment, on one hand, and depression and perceived sense of discrimination (significant moderate correlations), on the other. Length of residence positively correlated with both types of adjustment and language fluency, and negatively correlated with depression. Depression, in turn, positively correlated with perceived sense of discrimination. Willingness to interact with host culture, on the other hand, negatively correlated with the perceived sense of discrimination and positively correlated with the fluency of host language.

### 3.3 Country-specific samples

Descriptive data of the country-specific samples varied significantly. The only variable that did not produce statistically significant differences among three subgroups was perceived sense of discrimination. Average length of residence was highest in Greece, while average age was significantly lower in Germany compared to the other two countries. The German subsample also showed highest language fluency rates at both stages, highest rates of psychological adjustment and willingness to interact with host nationals, highest percentage of individuals with good financial standing, and the lowest numbers of unemployment and history of being undocumented. The Greek subsample, on the other hand, showed highest rate of sociocultural adjustment, while the Italian subgroup reported highest rate of depression and lowest rates of both sociocultural and psychological adjustment (see Tables 3, 4). Overall, the subsamples of Greece and Italy showed more similarities, while the German subgroup notably differed.

**Table 3:** Country-specific sample mean scores.

	Italy	Greece	Germany	*F*	*p*
1 Length of residence	8.25	**14.50**	9.51	15.68	0.000
2 Age	**47.90**	**49.88**	36.93	75.90	0.000
3 Fluency of host language upon relocation	1.37	1.37	**2.34**	48.02	0.000
4 Fluency of host language now	3.54	**4.00**	**4.26**	31.42	0.000
5 Frequency of visiting Georgia	2.41	2.35	**3.34**	27.76	0.000
6 Perceived sense of discrimination	1.91	2.05	2.01	0.65	0.000
7 Depression	**2.27**	**2.21**	1.93	12.18	0.000
8 Willingness to interact with host nationals	3.56	**3.68**	**3.80**	3.90	0.000
9 Sociocultural adjustment	3.67	**3.89**	**3.78**	3.10	0.000
10 Psychological adjustment	2.85	3.11	**3.44**	22.52	0.000

Significant differences are shown in bold.

**Table 4:** Country-specific sample frequency distributions.

		Italy	Greece	Germany	*χ*^2^	*p*
Financial/income State	Insufficient	9.00%	14.70%	3.00%	67.41	0.000
	Sufficient only for basic needs	34.20%	30.70%	15.90%		
	Satisfactory	49.00%	46.60%	44.30%		
	Good	6.50%	8.00%	31.30%		
	Excellent	1.30%	0.00%	5.50%		
History of marriage with host national	No	95.50%	82.70%	76.10%	24.86	0.000
	Yes	4.50%	17.30%	23.90%		
History of illegal immigration	No	48.40%	50.70%	91.50%	90.18	0.000
	Yes	51.60%	49.30%	8.50%		
Employment	Full-time	69.70%	62.70%	54.20%	51.15	0.000
	Part-time	11.60%	10.70%	12.40%		
	Student	1.90%	1.30%	15.40%		
	Housewife	9.00%	17.30%	9.00%		
	Retired/other	0.00%	2.70%	7.00%		
	Unemployed	7.10%	5.30%	2.00%		

### 3.4 Hypothesis testing

Further, we performed regression analyses to test hypotheses. The results showed that our hypotheses were partially confirmed (see Table 5).

**Table 5:** Regressions.

	Model		Coefficients		
	*R*^2^	Sig. *F* change	*β*	*t*	Sig.
**Predictors of depression**	0.24	0.000			
Fluency of host language upon relocation			0.13	2.47	0.014
History of illegal immigration status			0.12	2.50	0.013
Perceived sense of discrimination			0.15	2.95	0.003
Sociocultural adjustment			−0.28	−5.36	0.000
**Predictors of psychological adjustment**	0.45	0.000			
Age			−0.20	−4.06	0.000
Fluency of host language now			0.11	2.08	0.039
Low income			−0.09	−2.21	0.028
Perceived sense of discrimination			−0.29	−6.66	0.000
Willingness to interact with host nationals			0.11	2.57	0.011
Sociocultural adjustment			0.29	6.68	0.000

Depression was positively predicted by perceived sense of discrimination and history of illegal immigration, also surprisingly with the fluency of host language upon relocation, and was negatively predicted by sociocultural adjustment. The latter was the strongest predictor of depression. The model is significant: *F*(12, 374) = 9.760, *p* < 0.000 and explains 24% of variance in depression.

Psychological adjustment was positively predicted by sociocultural adjustment, willingness to socialize with host nationals, and language fluency at the time of the study, while age, perceived sense of discrimination, and poor financial state acted as negative predictors. Sociocultural adjustment was again the strongest predictor along with the perceived sense of discrimination. The model is significant: *F*(12, 374) = 25.504, *p* < 0.000; 45% of variance in psychological adaptation is explained.

## 4 Discussion

### 4.1 Demographic variables and acculturation conditions

The sample’s overall scores suggested a healthy tendency towards adaptation of Georgian migrants in EU countries as the mean scores of language fluency and adaptive outcomes (e.g., psychological and sociocultural adjustment) were all above midpoint and the mean scores of maladaptive outcomes (e.g., depression, discrimination)—below midpoint. Considering that average lengths of residence of the sample neared 10 years, the study descriptive statistics confirmed that during cross-cultural transition most people eventually adapt to a new culture (Ward and Kus, 2012; Berry and Hou, 2014).

Regarding intercultural distance, the differences in our subsamples in regards with the adaptation variables did not mirror the differences reflected in cultural compass with the only exception of sociocultural adjustment being highest in Greece. Overall, the psychological well-being outcomes were the most favorable among the German sample—the county with the largest cultural distance. These results, however, did not in any way devalue the cultural proximity factor, instead they pinpointed the superior power of acculturation conditions over cultural proximity, particularly with younger sample. In modern digital era cultural boundaries tend to become less distinct, especially for younger generations.

While the Greece and Italy subgroups, for the most part, presumably consisted of middle-aged female labor migrants involved in emotionally draining domestic jobs (e.g., taking care of the elderly), German sample stood out as considerably (12 years) younger, with highest number of students, better language fluency upon relocation, highest number of marriages with host nationals, and lowest percentages of unemployment and illegal immigration history. These differences may explain German sample’s better adaptation and well-being outcomes. Among relatively similar subgroups of Greece and Italy, however, the one with higher cultural proximity showed considerably higher adjustment outcomes (both sociocultural and psychological) and lower depression despite somewhat inferior financial standing. Thus, cultural distance seems to matter especially with older migrants; yet, it can be overshadowed by host country conditions and may not be as critical for younger Georgians who, per other evidence, exhibit growing tendency towards individualism and westernization (Skhirtladze et al., 2016; Shekriladze et al., 2021). More studies are warranted to examine the role of intercultural distance in diverse contexts and its links with adaptation of Georgian immigrants.

### 4.2 Notable associations and predictions

Unlike some other studies, length of residence did not act as statistically significant predictor although it positively correlated with both types of adjustment and negatively correlated with depression thereby confirming the trajectory of most people eventually adapting to a new culture. Age, on the other hand, negatively predicted psychological adjustment suggesting that cross-cultural transition is easier for younger Georgians.

In line with other evidence from the US and EU (Meghani and Harvey, 2016; Garcini et al., 2017, 2021; Fakhoury et al., 2021; Refle et al., 2023), history of illegal immigration and unfavorable financial state were linked with depression and poorer psychological adjustment, whereas perceived sense of discrimination acted as a valuable predictor of both depression and inferior psychological adaptation. These findings were consistent with other evidence reporting negative associations between perceived discrimination and well-being (Ozer, 2015; Adger, 2016; Schwartz et al., 2018; Mougenot et al., 2021).

Furthermore, our results showed that language fluency at the time of study and desire to interact with host nationals do not relate to depression; yet they both represented important ingredients of better psychological adjustment. Similar to our findings, many studies singled out fluency in host language and connections with host nationals as predictors of better adaptation and acculturation (Yang et al., 2006; Miglietta and Tartaglia, 2009; Ozer, 2015; Chang, 2016; Darmanaki Farahani and Bradley, 2018; Bethel et al., 2020; Nakhaie, 2020; Razgulin et al., 2023).

Nevertheless, our findings showed that language fluency upon relocation positively predicted depression. This might suggest that those with good command of host language might have had higher expectations (e.g., about finding a job) which were not necessarily met, or might have found themselves prematurely indulged in a new culture, both conditions potentially contributing to their increased vulnerability. Alternatively, it may also pinpoint that knowing the language makes individuals more aware of surroundings, particularly while interacting with host nationals. Studies show that immigrants for whom the host language is a second language tend to encounter prejudice, neglect or social rejection from native speakers (Lee and Rice, 2007; Lippi-Green, 2012). At the initial stage of relocation, on one hand, people tend to be more sensitive and, on the other hand, the chances of cultural misunderstandings are higher. Thus, linguistic competence might make individuals more susceptible to negativity, which in turn may impact their mood.

The absence of association between depression and host-language fluency, on the other hand, may suggest that interaction with members of original culture is critical, particularly at the initial stages of relocation, and may prevent immigrants from developing depression. A study from Spain showed that having social support from native community was associated with better immigrant mental health (Hombrados-Mendieta et al., 2019). In today’s globalized digital world, immigrant populations obviously enjoy more opportunities to connect with members of their culture of origin, which might serve as a buffer from acculturative stress.

To feel well-adjusted in a host country, however, avoiding depression does not seem to be sufficient. Our results suggested that in addition to sociocultural adjustment, feeling equal (no sense of perceived discrimination) and connected (willingness to interact) to mainstream culture is imperative for which fluency in host language is crucial. With language competence immigrants become able to build social connections and support network in a host culture—all powerful predictors of better adjustment (Guruge et al., 2015; Bethel et al., 2020; Pinamang et al., 2021; Mohanan, 2022). Thus, willingness to interact with host nationals coupled with the proficiency in a mainstream language in the absence of discriminative experiences produces better prospects for adaptation.

Finally, envisaging sociocultural adjustment as a predictor variable generated valuable insights. Sociocultural adjustment entailed individual experience of adaptation to various life domains such as climate, nature, language, social environment, practicalities, food, people, and social norms among others. According to our findings, sociocultural adjustment acted as the most important determinant of migrants’ better psychological well-being strongly predicting both lower depression and higher psychological adaptation. Further analysis entails examining the potential mediating roles of various variables (e.g., language fluency, perceived discrimination, history of being undocumented, etc.) with respect to sociocultural adjustment.

Overall, our study produced valuable findings on migration-related matters singling out the role of sociocultural adjustment in migrant adaptation. The results confirmed previous evidence on the links between the number of variables and well-being outcomes, and generated new insights on less expected link between depression and host-language fluency upon relocation, on one hand, and interrelationships between the contextual factors and intercultural distance, on the other. These findings highlighted a multifaceted and complex nature of migratory experience and call for further evidence.

In sum, our results suggested that immigrant adaptation to a new country is a very complex process in which multiple factors play integral part. Interdisciplinary approach to examining and solving migration-related complexities has to be favored. More research is needed to obtain better insight on ways of promoting migrant resilience.

## 5 Limitations

Major limitations of the study include sampling bias and subsample differences as well as predominance of female participants (partly explained by feminization of labor market in two study countries) that create challenges for generalizability of the findings. Additional limitations entail the possibility of inaccurate completions and challenges associated with self-report measures and e-surveys, for all their advantages, as opposed to face-to-face research, in reaching geographically scattered samples. Furthermore, the study could not examine all important acculturation conditions (e.g., leaving family members behind) that may have impacted participants’ mental well-being. Finally, we recognize the limits of psychological approach of our research that does not fully examine a myriad of systemic characteristics, particularly of novel social environment (e.g., immigration policies and contexts of the receiving country, etc.) relevant to immigrant adaptation.

## 6 Conclusion

Our results suggested that adjustment in diverse sociocultural domains of life was the most critical for immigrants’ psychological well-being along with the lack of perceived discrimination. Younger age, willingness to interact with the mainstream culture, fluency in host language, better financial standing and no history of illegal immigration stood out as additional factors associated with better adjustment and well-being outcomes. The results also pinpointed that language proficiency at the initial stage of relocation may contribute to migrant susceptibility and intercultural distance may be overshadowed by acculturation conditions in importance. The findings, thus, emphasized the complexity of migration and culture change and pinpointed the advantages of deploying integrated wholistic policies and practices to promote smooth transition of immigrant populations via support in various life domains.

## Data availability statement

The raw data supporting the conclusions of this article will be made available by the authors, without undue reservation.

## Author contributions

IS conceptualized the research, developed the study questionnaire, led the data collection, performed the data analysis part and interpretation, and wrote the manuscript. NJ participated in research planning and conceptualization, carried out data processing and analysis, and contributed to the final manuscript. All authors contributed to the article and approved the submitted version.
